# Design, synthesis and biological evaluation of hybrids of β-carboline and salicylic acid as potential anticancer and apoptosis inducing agents

**DOI:** 10.1038/srep36238

**Published:** 2016-11-08

**Authors:** Qi-Bing Xu, Xiang-Fan Chen, Jiao Feng, Jie-Fei Miao, Ji Liu, Feng-Tao Liu, Bi-Xi Niu, Jin-Yang Cai, Chao Huang, Yanan Zhang, Yong Ling

**Affiliations:** 1 School of Pharmacy and Jiangsu Province Key Laboratory for Inflammation and Molecular Drug Target, Nantong University, Nantong 226001, China; 2 State Key Laboratory of Natural Medicines, China Pharmaceutical University, Nanjing 210009, P.R. China; 3 Tumor chemotherapy Department, Affiliated Hospital of Nantong University, China

## Abstract

A novel series of hybrids (7a-l, 8a-l) from β-carboline and salicylic acid (SA) were designed and synthesized, and their *in vitro* biological activities were evaluated. Most of the hybrids displayed potent antiproliferative activity against five cancer cell lines *in vitro*, showing potencies superior to 5-FU and harmine. In particular, compound 8h selectively inhibited proliferation of liver cancer SMMC-7721 cells but not normal liver LO2 cells, and displayed greater inhibitory selectivity than intermediate 5h and SA. 8h also induced cancer cell apoptosis in an Annexin V-FITC/propidium iodide flow cytometry assay, and triggered the mitochondrial/caspase apoptosis by decreasing mitochondrial membrane potential which was associated with up-regulation of Bax, down-regulation of Bcl-2 and activation levels of the caspase cascade in a concentration-dependent manner. Our findings suggest that the β-carboline/SA hybrids may hold greater promise as therapeutic agents for the intervention of human cancers.

Natural products have been a rich source of compounds for drug discovery and the majority of anticancer drugs currently used in clinical are derived from natural product scaffolds. β-Carbolines alkaloids, a family of natural products possessing common parent nucleus of a planar tricyclic pyrido[3,4-b]indole ring system, are abundant in the plant kingdom or other organisms^1^. Some β-carboline derivatives, both natural and synthetic compounds such as harmine, harmane, norharman, and β-carboline-benzimidazole conjugates (Fig. 1), have been reported to display antitumor activities through multiple mechanisms such as intercalating into DNA or inhibiting Topoisomerase I and II, cyclin-dependent kinases (CDK), PLK, or MAO^2^,^3^,^4^,^5^,^6^. According to the SARs summarized from the reported β-carboline derivatives, the modification on positions-1 or -3 of the parent nucleus would make great influence on their antitumor activities^6^,^7^,^8^,^9^.

Many natural and synthetic anticancer agents with the ability to interact with DNA, including β-carbolines, often have relatively low therapeutic index, most likely due to the unspecific manner of these agents in causing DNA damage both in neoplastic and in highly proliferative normal tissues^10^. Hybrid molecules, which combine structural motifs from two or more biologically active agents, often have improved potency and/or reduced toxicity, and have received increased attention recently. A variety of hybrids have been reported to date that displayed improved anti-tumor activities than the individual agents^2^,^9^,^11^,^12^,^13^.

Recently, we have shown that hybrids of β-carboline and hydroxamic acid showed synergistic effects and displayed increased anticancer activity^9^. In this manuscript, we report the design and synthesis of hybrid compounds featuring structurally moieties of β-carboline and acetylsalicylic acid (aspirin). Aspirin, a well known nonsteroidal anti-inflammatory agent, has been revealed to exhibit high potency in the treatment of cancer^14^,^15^,^16^. Epidemiological studies suggested that the regular intake of aspirin was associated with a reduction in the incidence of malignancies, including colorectal, gastrointestinal, and lung cancer^17^,^18^. Recent studies demonstrated that aspirin and its metabolite salicylic acid (SA), and their derivatives could induce apoptosis in several colorectal carcinoma cell lines^19^,^20^,^21^,^22^.

A series of novel β-carbolines/salicylic acid hybrids **7a-l** and **8a-l** have been designed by introducing the salicylic acid fragment into the β-carboline molecule at the 3-position with diamines linkers of different length. We hypothesized that the novel types of hybrids could selectively inhibit tumor cell proliferation and efficaciously induce tumor cell apoptosis with a synergy for the treatment of cancer. In addition, given the importance of the 1-position of the β-carboline for its antitumor activity, hybrids bearing different substituents at this position were also investigated. Therefore, twenty β-carbolines/salicylic acid hybrids were designed and synthesized, and their *in vitro* antitumor activities were evaluated. Herein, the synthesis and biological evaluation of these compounds were reported.

## Results and Discussion

### Chemistry

**Figure 2:**
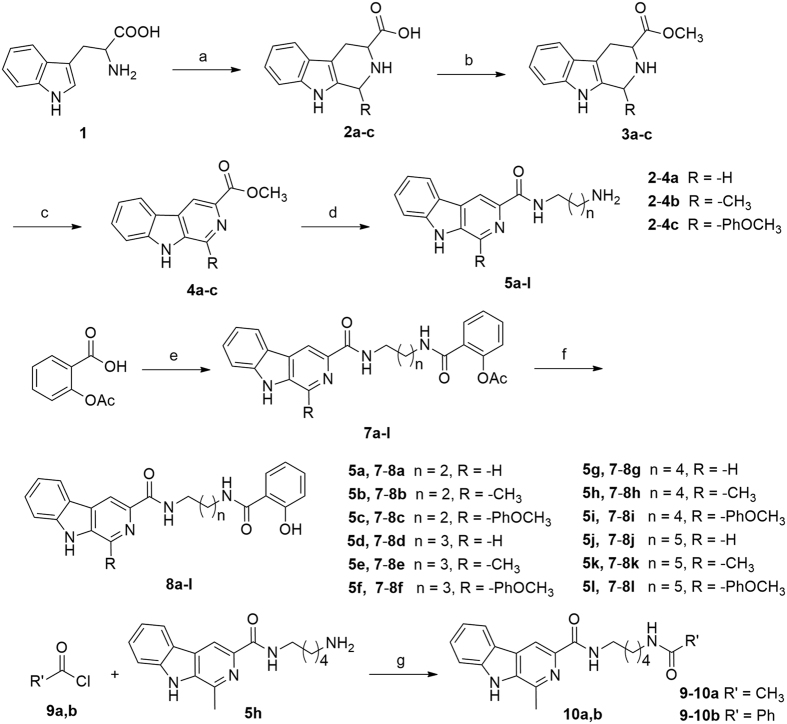
Reagents and conditions. (**a**) H^+^ or OH^−^, RCHO, reflux, 2–4 h, 81–86%; (**b**) SOCl_2_, MeOH, 0 °C, 1 h, and then reflux 6 h, 90–95%; (**c**) KMnO_4_, DMF, reflux, 6 h, 60–70%; (**d**) different diamines, EtOH, reflux, 3–5 h, 81–89%; (**e**) i) *N*-methylmorpholine, ethyl chloroformate, THF, 0 °C, 1 h; ii) Et_3_N, THF, 0 °C, 1–3 h, 55–68%; (**f**) NaOH, MeOH, r.t., 2 h, 90–96%; (**g**) triethylamine, CH_2_Cl_2_, r.t., 2 h, 75–79%.

The synthetic route to compounds **7a-l, 8a-l** is depicted in Fig. 2. The substituted β-carbolines **4a-c** were prepared in a three-step sequence. First, commercially available L-tryptophan **1** was converted to 1-substituted-1,2,3,4-tetrahydro-β-carboline-3-carboxylic acid **2a-c** in a Pictet-Spengler reaction using differently substituted aldehydes (formaldehyde, acetaldehyde or 4-methoxyphenaldehyde). Second, methyl esterification of compounds **2a-c** using methanol and SOCl_2_ afforded compound **3a-c**. Intermediates **3a-c** were oxidized by KMnO_4_ in DMF to afford compounds **4a-c**, which were then reacted with different diamines in EtOH to provide amides **5a**-**l**. Reaction of acetylsalicylic acid **6** with **5a**-**l** in the presence of ethyl chloroformate and *N*-methylmorpholine yielded compounds **7a-l**. Finally, hydrolysis of **7a-l** afforded compounds **8a-l**. The acetamide **10a** and benzamide **10b** were prepared by the treatment of **5h** with acetyl chloride **9a** or benzoyl chloride **9b**, respectively, in the presence of triethylamine. All target compounds were purified by column chromatography, and their structures were confirmed by IR, ^1^H NMR, MS, HRMS, and elemental analyses.

### Biological evaluation and structure-activity relationships (SARs) analysis

The *in vitro* antitumor activities of the synthesized compounds **7a-l** and **8a-l** against five human cancer cells were evaluated in MTT assays using 5-fluorouracil (5-FU) and harmine as positive controls. These cells are human hepatocellular carcinoma cells (SMMC-7721 and Hep G2), human colon cancer cell lines (HCT116), human bladder carcinoma cells (EJ), and human lung cancer cells (H460). Their IC_50_ values (the effective concentration that inhibiting 50% of tumor cell proliferation) are listed in Table 1.

Most of the synthesized compounds exhibited higher inhibitory potency than 5-FU and harmine in all five cell lines. For instance, **7g-h**, **7k**, **8d-e**, **8g-h**, and **8k** displayed anti-proliferation activities in the low micromolar range of 6.97–19.4 μM, which were significantly greater than the parent compound harmine. In particular, **8h** showed the greatest inhibition activities for liver and colon cancer cells growth with IC_50_ values of 6.97–8.25 μM, which was 4~6 fold lower than that of 5-FU (IC_50_ = 19.6–35.2 μM). Importantly, when the salicylamide was replaced with either an acetamide (**10a**) or benzamide **(10b**), the antitumor activities were significantly weaker (>50 μM), indicating the contribution of the salicylamide structural moiety in **8h** to its antitumor activity and the improved anti-proliferation potency.

Analysis of SAR reveals that the length of hybrids linkers influences the antitumor activities. Consistent with previous hybrid compounds, the potency first increases then decreases with the elongation of the linker. For example, hybrids linked with butanediamine (n = 3) and amyl diamine (n = 4) exhibited greater antitumor activities than that with propane diamine (n = 2) or hexanediamine (n = 5). Compounds with a methyl group at the 1-position of the β-carboline showed greater potency than the other two classes containing either hydrogen or *p*-methoxyphenyl (e.g. **8h** vs. **8g** vs. **8i**; **7h** vs. **7g** vs. **7i**). The potency order is methyl >hydrogen >*p*-methoxyphenyl for substitutes at the 1-position of the β-carboline. These results suggest that an appropriate substitution is preferred at the 1-position for antitumor activities of these hybrids. Interestingly, compounds **8a-l** in general displayed greater antitumor activity than their acetylated compounds **7a-l**, suggesting the phenolic hydroxyl group is preferred for antitumor activities.

**Figure 3:**
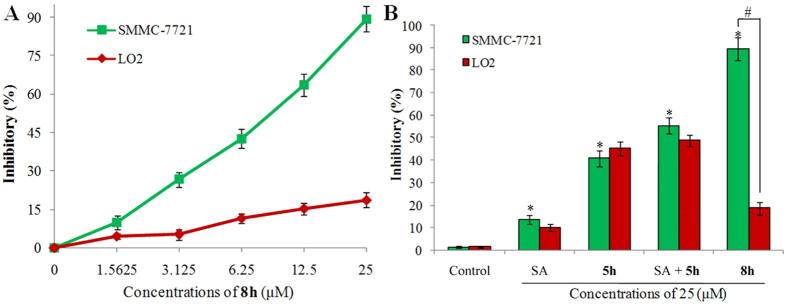
(**A**) Inhibitory effects of **8h** on the proliferation of SMMC-7721 and LO2 cells. Cells were incubated with the indicated concentrations of **8h** for 48 h. Cell proliferation was assessed using the MTT assay. Data are means ± SD of the inhibition (%) from three independent experiments. (**B**) Inhibitory effects of **5h**, **8h**, and SA or the vehicle control against SMMC-7721 and LO2 cells. SMMC-7721 and LO2 cells were incubated with the indicated compounds at 25 μM for 48 h, and cell proliferation was assessed by the MTT assay. Data are means ± SD of the inhibition (%) from three independent experiments. *P < 0.01 versus control of SMMC-7721, ^#^*P* < 0.01 inhibitory effects of **8h** in SMMC-7721 versus LO2 cells.

Given the strong growth inhibitory activity of **8h** *in vitro*, it was profiled for antitumor selectivity by examining its inhibitory effects on the growth of SMMC-7721 cells and LO2 cells (human hepatocellular normal cells). The dose-response curve is shown in Fig. 3A. As can be seen, no apparent growth inhibitory activity was observed on the non-cancer LO2 cells at concentrations up to 25 μM. However, **8h** displayed apparent anti-proliferation activity on SMMC-7721 cells with IC_50_ of 6.97 μM, which is 9 fold lower than against liver normal LO2 cells (IC_50_ = 55.2 μM), confirming that **8h** can selectively suppress the proliferation of tumor cells *in vitro*.

The inhibitory activity of **8h**, SA, and **5h,** an analog of **8h** without the SA structural moiety, against SMMC-7721 cells and LO2 cells were evaluated at a concentration of 25 μM for 48 h. Figure 3B shows that treatment with SA alone induced little proliferation of both SMMC-7721 and LO2 cells, similar to those of the cells treated with vehicle. Treatment with **5h** caused much greater inhibitory effects (~42%) against SMMC-7721 cells than SA. Compound **5h (**IC_50_ = 27.2 ± 3.4 μM) was also more potent in inhibiting SMMC-7721 cell growth than the two “capped” analogs **10a** (IC_50_ = 58.6 ± 6.7 μM) or **10b** (IC_50_ > 100 μM), in which the primary amino group was acylated to afford either an acetamide or a benzamide. However, the inhibitory effect of **5h** appears to be non-specific for tumor cells, as a similar effect was seen in the non-tumor LO2 cells. Such an increase on both potency and cytotoxicity with the introduction of an alkyl amino group is consistent with previous reports^23^,^24^, and is likely the result of non-specific interactions between the primary amino group in **5h** (mostly protonated at pH 7.4) and residues around the carboline binding site in both cell types. At 25 uM, hybrid **8h** displayed significant inhibitory effects on tumor cell proliferation (~90%). This is markedly greater than SA (~15%) and carboline analog **5h** (~42%), and also greater than the combination of SA and **5h** (~55%, Fig. 3B). Given that the inhibitory activity of **8h** is significantly higher than SA, it is likely that the β-carboline moiety may provide greater contribution to the overall antitumor activity. These results suggest that the increased anti-tumor activity of **8h** most likely result from the presence of both the β-carboline and SA moieties in **8h**, suggesting the existence of the synergetic effects by these two agents.

Importantly, **8h** showed little inhibitory effects on the LO2 cells, in contrast to **5h** which had high toxicities to these normal cells. These results indicate that the SA structural moiety in **8h** contributes to the improved anti-proliferation potency and reduced toxicity (**8h** vs **5h**), which therefore represents a more potent and safer antitumor agent than β-carboline alone, which may warrant further investigation and development.

**Figure 4:**
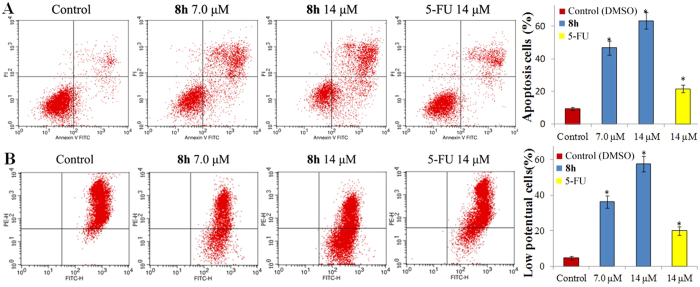
Compound 8h induced SMMC-7721 cell apoptosis *in vitro*. SMMC-7721 cells were incubated with the indicated concentrations of **8h** and 14 μM 5-FU for 48 h, and the cells were stained with FITC-Annexin V and PI, followed by flow cytometry analysis. (**A**) Flow cytometry analysis. (**B**) Quantitative analysis of apoptotic cells. Data are expressed as means ± SD of the percentages of apoptotic cells from three independent experiments. *P < 0.01 vs control.

To determine whether apoptosis is involved in the anti-proliferative effects of **8h**, apoptosis assay was performed using SMMC-7721 cells. The cells were incubated with either vehicle alone, different concentrations of **8h** (7.0 or 14 μM), or 14 μM 5-FU for 48 h, and then stained with FITC-Annexin V and propidium iodide (PI). The percentages of apoptotic SMMC-7721 cells were determined by flow cytometry analysis. The results are shown in Fig. 4A. In the vehicle group, the occurrence of SMMC-7721 cell apoptosis was minimal. However, in the **8h**-treated SMMC-7721 cells, the population of the apoptosis cells clearly increased. Low concentration of **8h** (7.0 μM) induced 46% SMMC-7721 cell apoptosis, while the higher dose (14 μM) of **8h** induced 63% SMMC-7721 cell apoptosis. Both are significantly greater than those induced by 5-FU at 14 μM (only 22%). These results revealed that the antitumor activity of **8h** is associated with the apoptosis effect which appeared to be a concentration-dependent manner.

The mitochondrial pathway plays an important role in cell apoptosis^25^. To determine whether mitochondria is involved in the apoptosis-inducing effect of **8h**, changes in mitochondrial membrane potential were investigated with a fluorescent probe JC-1. JC-1 is a lipophilic cationic dye which facilitates the penetration of mitochondrial membrane due to the decrease of membrane potential, indicated by a fluorescence shift from red to green. The cells were treated with vehicle alone, with different concentrations of **8h** (7.0 or 14 μM), or 14 μM 5-FU for 48 h, and then stained with JC-1. As shown in Fig. 4B, in the control group, the percentage of cells emitting green fluorescence was only 4.1%, whereas cells treated with **8h** exhibited stronger green fluorescence. The percentage of cells emitting green fluorescence reached 57% at the higher dose of 14 μM. The observations indicate that **8h** plays a role in the dissipation of mitochondrial membrane potential and the apoptosis effect of **8h** is associated with mitochondrial depolarization in cells.

**Figure 5:**
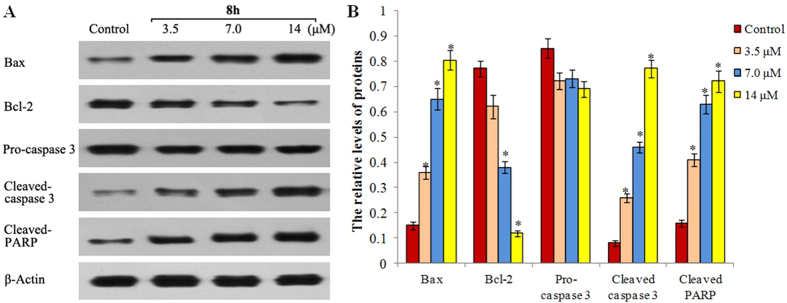
Effect of 8h on the expression of apoptosis-related proteins in SMMC-7721 cells. (**A**) The expression of Bax, Bcl2, caspase 3 and β-actin was examined by western blot analysis. SMMC-7721 cells were incubated with, or without, **8h** at the indicated concentrations for 48 h and the levels of protein expression were detected using specific antibodies, and β-actin was used as the control. Data shown are representative images of each protein for three separate experiments. (**B**) Quantitative analysis: the relative levels of each protein compared to control β-actin were determined by densimetric scanning. Data are expressed as means ± SD from three separate experiments. *P < 0.01 vs control.

Next, to investigate the preliminarily molecular mechanisms underlying the cell apoptosis profiles in **8h**-treated cells, we conducted western blot analysis to examine the expression of apoptosis proteins in SMMC-7721 cells. It is well known that Bcl-2 and Bax are anti-apoptotic or pro-apoptotic regulator proteins, respectively, and caspase-3 is the execution factor of apoptosis. In addition, PARP is one of several known cellular substrates for caspase-3 and the cleavage of PARP by caspase-3 is considered to be a hallmark of apoptosis^26^. As shown in Fig. 5, the protein levels of Bax proteins significantly increased in **8h**-treated cells, whereas the expression of Bcl-2 was dramatically reduced in a concentration-dependent manner. Moreover, compound **8h** resulted in more significant cleavage of both PARP and caspase-3 than the control group. These results indicate that **8h** could significantly induce cell apoptosis through the regulation of apoptosis proteins.

## Conclusions

A novel series of hybrids (**7a**-**l**, **8a−l)** from β-carboline and SA were synthesized, and their *in vitro* biological activities were evaluated. It was found that most of the hybrids showed potent anti-proliferation activities against five human cancer cells *in vitro*. The most potent compound **8h** exhibited the highest inhibition activities against liver and colon cancer cells with IC_50_ values of 6.97–8.25 μM which greater than that of 5-FU (IC_50_ = 19.6–35.2 μM), and selectively inhibited cancer cells but not non-tumor liver cell proliferation *in vitro*. Furthermore, **8h** dose-dependently induced cancer cell apoptosis which is associated with mitochondrial depolarization in tumor cells by up-regulating Bax, down-regulating Bcl-2, in addition to activating levels of the caspase cascade in a concentration-dependent way. Therefore, our novel findings provide a proof of principle in the design of new β-carboline/SA hybrids for the intervention of human cancers.

## Methods

### Chemistry

Infrared (IR) spectra (KBr) were recorded on a Nicolet Impact 410 instrument (KBr pellet). ^1^H NMR spectra were recorded with a Bruker Avance 300 MHz spectrometer at 300 K, using TMS as an internal standard. MS spectra were recorded on a Mariner Mass Spectrum (ESI). High resolution mass spectra were recorded using an Agilent Technologies LC/MSD TOF. Element analysis was performed on an Eager 300 instrument. All compounds were routinely checked by TLC and ^1^H NMR. TLCs and preparative thin-layer chromatography were performed on silica gel GF/UV 254, and the chromatograms were conducted on silica gel (200–300 mesh, Merck) and visualized under UV light at 254 and 365 nm. All solvents were reagent grade and, when necessary, were purified and dried by standards methods. L-tryptophan **1**, acetylsalicylic acid **6** and different substituted aldehydes were commercially available. Compounds **2a-c**, **3a-c**, and **4a-c** were synthesized according literatures^2^,^7^,^9^. Solutions after reactions and extractions were concentrated using a rotary evaporator operating at a reduced pressure of ca. 20 Torr. Organic solutions were dried over anhydrous sodium sulfate. High-performance liquid chromatography (HPLC) analysis conditions: column: Shimadzu C18 (150 mm × 4.6 mm × 5 μm); mobile phase: methanol: water = 75: 25; wavelength: 254 nm; flow rate: 1 mL/min. All target compounds were of >95% purity as determined by HPLC.

### *N*-(3-Aminopropyl)-9H-pyrido[3,4-b]indole-3-carboxamide(5a)

A mixture of **4a** (2.3g, 10 mmol) and 1,3-propanediamine 8.3 mL (100 mmol) in 15 mL methanol was refluxed for 10 h. After cooled, the solvent was evaporated. The residue was purified by silica chromatography (CH_2_Cl_2_-CH_3_OH = 6:1, v/v as the eluent) to afford a yellowish solid 2.3 g, yield: 85%. MS (ESI) m/*z* = 269 [M+H]^+^.

### *N*-(3-Aminopropyl)-1-methyl-9H-pyrido[3,4-b]indole-3-carboxamide(5b)

Refer to the synthesis of **5a**, the title product was obtained from **4b** and 1,3-propanediamine to afford a pale yellow solid 2.3 g, yield: 83%. MS (ESI) *m*/*z* = 283 [M+H]^+^.

### *N*-(3-Aminopropyl)-1-(4-methoxyphenyl)-9H-pyrido[3,4-b]indole-3-carboxamide(5c)

Refer to the synthesis of **5a**, the title product was obtained from **4c** and 1,3-propanediamine to afford a pale yellow solid 3.1 g, yield: 82%. MS (ESI) *m*/*z* = 375 [M+H]^+^.

### *N*-(4-Aminobutyl)-9H-pyrido[3,4-b]indole-3-carboxamide(5d)

Refer to the synthesis of **5a**, the title product was obtained from **4a** and 1,4-diaminobutane to afford the pale yellow solid 2.4 g, yield: 87%. MS (ESI) *m*/*z* = 283 [M+H]^+^.

### *N*-(4-Aminobutyl)-1-methyl-9H-pyrido[3,4-b]indole-3-carboxamide(5e)

Refer to the synthesis of **5a**, the title product was obtained from **4b** and 1,4-diaminobutane to afford the pale yellow solid 2.5 g, yield: 84%. MS (ESI) *m*/*z* = 297 [M+H]^+^.

### *N*-(4-Aminobutyl)-1-(4-methoxyphenyl)-9H-pyrido[3,4-b]indole-3-carboxamide(5f)

Refer to the synthesis of **5a**, the title product was obtained from **4c** and 1,4-diaminobutane to afford the pale yellow solid 3.1 g, yield: 81%. MS (ESI) *m*/*z* = 389 [M+H]^+^.

### *N*-(5-Aminopentyl)-9H-pyrido[3,4-b]indole-3-carboxamide(5g)

Refer to the synthesis of **5a**, the title product was obtained from **4a** and 1,5-pentanediamine to afford the pale yellow solid 2.6 g, yield: 87%. MS (ESI) *m*/*z* = 297 [M+H]^+^.

### *N*-(5-Aminopentyl)-1-methyl-9H-pyrido[3,4-b]indole-3-carboxamide(5h)

Refer to the synthesis of **5a**, the title product was obtained from **4b** and 1,5-pentanediamine to afford the pale yellow solid 2.6 g, yield: 84%. ^1^H NMR (DMSO-*d*_6_, 300 MHz): *δ* 8.66 (m, 1H, Ar-H), 8.59 (m, 1H, NH), 8.33 (d, 1H, *J* = 6.0 Hz, Ar-H), 7.55–7.63 (m, 2H, Ar-H), 7.28 (m, 1H, Ar-H), 3.36 (m, 2H, NCH_2_), 2.84 (s, 3H, ArCH_3_), 2.57 (m, 2H, NCH_2_), 1.42–1.60 (m, 4H, 2 × NCH_2_CH_2_), 1.15–1.22 (m, 2H, NHCH_2_CH_2_CH_2_); MS (ESI) *m*/*z* = 311 [M+H]^+^; HRMS (ESI): *m*/*z* calcd for C_18_H_23_N_4_O: 311.1872; found: 311.1887 [M+H]^+^.

### *N*-(5-aminopentyl)-1-(4-methoxyphenyl)-9H-pyrido[3,4-b]indole-3-carboxamide(5i)

Refer to the synthesis of **5a**, the title product was obtained from **4c** and 1,5-pentanediamine to afford the pale yellow solid 3.3 g, yield: 82%. MS (ESI) *m*/*z* = 403 [M+H]^+^.

### *N-*(6-Aminohexyl)-9H-pyrido[3,4-b]indole-3-carboxamide (5j)

Refer to the synthesis of **5a**, the title product was obtained from **4a** and 1,6-hexamethylene diamine to afford the pale yellow solid 2.7 g, yield: 87%. MS (ESI) *m*/*z* = 311 [M+H]^+^.

### *N*-(6-Aminohexyl)-1-methyl-9H-pyrido[3,4-b]indole-3-carboxamide (5k)

Refer to the synthesis of **5a**, the title product was obtained from **4b** and 1,6-hexamethylene diamine to afford the pale yellow solid 2.8 g, yield: 85.3%. MS (ESI) *m*/*z* = 325 [M+H]^+^.

### *N*-(6-Aminohexyl)-1-(4-methoxyphenyl)-9H-pyrido[3,4-b]indole-3-carboxamide (5l)

Refer to the synthesis of **5a**, the title product was obtained from **4c** and 1,6-hexamethylene diamine to afford the pale yellow solid 3.5 g, yield: 85%. MS (ESI) *m*/*z* = 417 [M+H]^+^.

### *N*-(3-(2-Methylbenzamido)propyl)-9H-pyrido[3,4-b]indole-3-carboxamide (7a)

To a solution of acetylsalicylic acid **6** (1.8 g, 10 mmol) in 20 mL anhydrous THF/DMF (v:v = 3:1) was added N-methyl morpholine (1.5 g, 15 mmol) under ice bath. After slowly added ethyl carbonochloridate (1.3 g, 12 mmol), the mixture was stirred at room temperature for 0.5 h, and then added dropwise to 10 mL anhydrous THF solution of **5a** (2.7 g, 10 mmol) and triethylamine (1.5 g, 15 mmol) at 0 °C. The mixture was continued reacted for 2 h and added 100 mL water, then extracted with ethyl acetate (3 × 50 mL). The combined organic layer was washed with brine, dried with Na_2_SO_4_, and concentrated in vacuo. The residue was purified by silica gel column chromatography (petroleum ether: ethyl acetate = 2:3, v/v) to afford a pale yellow solid 3.4 g, yield: 77%. Analytical data for **7a**: IR (KBr, cm^-1^): 3451, 2963, 1657, 1574, 1485, 1428, 1301, 1265, 1028. ^1^H NMR (CDCl_3_, 300 MHz): *δ* 11.21 (s, 1H, NH), 8.89 (s, 1H, Ar-H), 8.71 (s, 1H, Ar-H), 8.11–8.19 (m, 3H, CONH, Ar-H), 7.58–7.76 (m, 4H, Ar-H), 7.26–7.48 (m, 3H, Ar-H), 3.63 (m, 2H, NCH_2_). 3.52 (m, 2H, NCH_2_), 2.38 (s, 3H, CH_3_CO), 1.92 (m, 2H, CH_2_); MS (ESI) *m*/*z* = 446 [M+H]^+^; Anal. Calcd. for C_25_H_25_N_4_O_4_: C, 67.40; H, 5.66; N, 12.58; Found: C, 67.23; H, 5.77; N, 12.47.

### 1-Methyl-*N*-(3-(2-methylbenzamido)propyl)-9H-pyrido[3,4-b]indole-3-carboxamide (7b)

Refer to the synthesis of **7a**, the title product was obtained from **5b** and acetylsalicylic acid to afford a pale yellow solid 3.3 g, yield: 73%. Analytical data for **7b**: IR (KBr, cm^−1^): 3391, 2943, 1618, 1503, 1480, 1398, 1310, 1236, 1028. ^1^H NMR (CDCl_3_, 300 MHz): *δ* 9.36 (s, 1H, NH), 8.87 (s, 1H, CONH), 8.73 (s, 1H, Ar-H), 8.47 (s, 1H, CONH), 8.15 (m, 1H, Ar-H), 8.06 (m, 1H, Ar-H), 7.58–7.63 (m, 2H, Ar-H), 7.42–7.45 (m, 2H, Ar-H), 7.30 (m, 1H, Ar-H), 7.06 (m, 1H, Ar-H), 3.70 (m, 2H, CH_2_N), 3.56 (m, 2H, NHCH_2_), 2.83 (s, 3H, ArCH_3_), 2.35 (s, 3H, CH_3_CO), 1.92 (m, 2H, NHCH_2_CH_2_); MS (ESI) *m*/*z* = 460 [M+H]^+^; Anal. Calcd. for C_26_H_27_N_4_O_4_: C, 67.96; H, 5.92; N, 12.19; Found: C, 67.88; H, 6.08; N, 12.01.

### 1-(4-Methoxyphenyl)-*N*-(3-(2-methylbenzamido)propyl)-9H-pyrido[3,4-b]indole-3-carboxamide (7c)

Refer to the synthesis of **7a**, the title product was obtained from **5c** and acetylsalicylic acid to afford a pale yellow solid 3.8 g, yield: 71%. IR (KBr, cm^−1^): 3450, 2904, 1626, 1549, 1485, 1406, 1313, 1220, 1020. Analytical data for **7c**: ^1^H NMR (DMSO-*d*_6_, 300 MHz): *δ−*11.05 (s, 1H, NH), 8.75 (s, 1H, Ar-H), 8.35–8.41 (m, 2H, Ar-H, CONH), 8.19 (d, 2H, *J* = 7.5 Hz, Ar-H), 7.84 (m, 1H, Ar-H), 7.57–7.65 (m, 3H, Ar-H), 7.41–7.46 (m, 3H, Ar-H), 7.06 (d, 2H, *J* = 7.5 Hz, Ar-H), 3.89 (s, 3H, OCH_3_), 3.68 (m, 2H, CH_2_N), 3.55 (m, 2H, NCH_2_), 2.32 (s, 3H, CH_3_CO), 1.89 (m, 2H, NCH_2_CH_2_). MS (ESI)*−m*/*z* = 552 [M+H]^+^; Anal. Calcd. for C_32_H_31_N_4_O_5_: C, 69.68; H, 5.66; N, 10.16; Found: C, 69.59; H, 5.77; N, 10.01.

### *N*-(4-(2-Methylbenzamido)butyl)-9H-pyrido[3,4-b]indole-3-carboxamide (7d)

Refer to the synthesis of **7a**, the title product was obtained from **5d** and acetylsalicylic acid to afford a pale yellow solid 3.5 g, yield: 77%. Analytical data for **7d**: IR (KBr, cm^−1^): 3451, 2883, 1676, 1514, 1456, 1400, 1343, 1260, 1010. ^1^H NMR (CDCl_3_, 300 MHz): *δ* 10.87 (br, 1H, NH), 8.88 (s, 1H, Ar-H), 8.74 (s, 1H, Ar-H), 8.28 (s, 1H, CONH), 8.16 (m, 1H, Ar-H), 7.69–7.71 (m, 1H, Ar-H), 7.49–7.57 (m, 3H, Ar-H), 7.30–7.38 (m, 2H, Ar-H), 7.09 (m, 1H, Ar-H), 3.57 (m, 2H, NCH_2_), 3.28 (m, 2H, CH_2_N), 2.29 (s, 3H, CH_3_CO), 1.58–1.67 (m, 4H, 2 × NCH_2_CH_2_); MS (ESI) *m*/*z* = 460 [M+H]^+^; Anal. Calcd. for C_26_H_27_N_4_O_4_: C, 67.96; H, 5.92; N, 12.19; Found: C, 67.82; H, 6.06; N, 12.11.

### 1-Methyl-*N*-(4-(2-methylbenzamido)butyl)-9H-pyrido[3,4-b]indole-3-carboxamide (7e)

Refer to the synthesis of **7a**, the title product was obtained from **5e** and acetylsalicylic acid to afford a pale yellow solid 3.7 g, yield: 77%. Analytical data for **7e**: IR (KBr, cm^−1^): 3478, 2952, 1677, 1542, 1494, 1446, 1311, 1265, 1028. ^1^H NMR (CDCl_3_, 300 MHz): *δ* 10.65 (s, 1H, NH), 8.75 (s, 1H, Ar-H), 8.70 (s, 1H, CONH), 8.30 (s, 1H, CONH), 8.14 (d, 1H, *J* = 6.0 Hz, Ar-H), 7.73 (d, 1H, *J* = 6.0 Hz, Ar-H), 7.53 (m, 2H, Ar-H), 7.42 (m, 1H, Ar-H), 7.29 (m, 2H, Ar-H), 7.06 (m, 1H, Ar-H), 3.48–3.59 (m, 4H, 2 × NCH_2_), 2.80 (s, 3H, ArCH_3_), 2.31 (s, 3H, CH_3_CO), 1.71–1.85 (m, 4H, 2 × CH_2_); MS (ESI) *m*/*z* = 474 [M+H]^+^; Anal. Calcd. for C_27_H_29_N_4_O_4_: C, 68.48; H, 6.17; N, 11.83; Found: C, 68.37; H, 6.23; N, 11.99.

### 1-(4-Methoxyphenyl)-*N*-(4-(2-methylbenzamido)butyl)-9H-pyrido[3,4-b]indole-3-carboxamide (7f)

Refer to the synthesis of **7a**, the title product was obtained from **5f** and acetylsalicylic acid to afford a pale yellow solid 4.1 g, yield: 72%. Analytical data for **7f**: IR (KBr, cm^−1^): 3451, 2963, 1599, 1504, 1435, 1428, 1333, 1265, 1011. ^1^H NMR (CDCl_3_, 300 MHz): *δ* 8.76 (s, 1H, Ar-H), 8.33 (s, 1H, CONH), 8.16 (m, 1H, Ar-H), 7.89–7.92 (d, 2H, *J* = 9.0 Hz, Ar-H), 7.47–7.69 (m, 4H, Ar-H), 7.30–7.35 (m, 2H, Ar-H), 7.12 (d, 2H, *J* = 9.0 Hz, Ar-H), 7.00 (m, 1H, Ar-H), 3.88 (s, 3H, OCH_3_), 3.57–3.59 (m, 2H, NCH_2_), 3.44–3.46 (m, 2H, NCH_2_), 2.30 (s, 3H, COCH_3_), 1.69–1.77 (m, 4H, 2 × NHCH_2_CH_2_); MS (ESI) *m*/*z* = 566 [M+H]^+^; Anal. Calcd. for C_33_H_33_N_4_O_5_: C, 70.07; H, 5.88; N, 9.91; Found: C, 69.92; H, 5.97; N, 9.82.

### *N*-(5-(2-Methylbenzamido)pentyl)-9H-pyrido[3,4-b]indole-3-carboxamide (7g)

Refer to the synthesis of **7a**, the title product was obtained from **5g** and acetylsalicylic acid to afford a pale yellow solid 3.5 g, yield: 73%. Analytical data for **7g**: IR (KBr, cm^−1^): 3467, 2966, 1625, 1545, 1480, 1421, 1321, 1235, 1018. ^1^H NMR (CDCl_3_, 300 MHz): *δ* 12.45 (br, 1H, NH), 8.93 (s, 1H, Ar-H), 8.76 (s, 1H, Ar-H), 8.26 (s, 1H, CONH), 8.11 (m, 1H, Ar-H), 7.48–7.63 (m, 3H, Ar-H), 7.25–7.38 (m, 3H, Ar-H), 7.06 (m, 1H, Ar-H), 3.61 (m, 2H, NCH_2_). 3.50 (m, 2H, NCH_2_), 2.33 (s, 3H, CH_3_CO), 1.73–1.82 (m, 4H, 2 ×NCH_2_CH_2_), 1.30 (m, 2H, NCH_2_CH_2_CH_2_); MS (ESI) *m*/*z* = 474 [M+H]^+^; Anal. Calcd. for C_27_H_29_N_4_O_4_: C, 68.48; H, 6.17; N, 11.83; Found: C, 68.32; H, 6.32; N, 11.69.

### 1-Methyl-*N*-(5-(2-methylbenzamido)pentyl)-9H-pyrido[3,4-b]indole-3-carboxamide (7h)

Refer to the synthesis of **7a**, the title product was obtained from **5h** and acetylsalicylic acid to afford a pale yellow solid 3.6 g, yield: 75%. Analytical data for **7h**: IR (KBr, cm^−1^): 3441, 2963, 1651, 1574, 1485, 1427, 1299, 1261, 1011. ^1^H NMR (CDCl_3_, 300 MHz): *δ* 10.42 (s, 1H, NH), 8.76 (s, 1H, Ar-H), 8.12–8.21 (m, 2H, Ar-H, CONH), 7.87 (m, 1H, Ar-H), 7.48–7.65 (m, 3H, Ar-H), 7.19–7.32 (m, 3H, Ar-H), 3.56 (m, 2H, NCH_2_), 3.38 (m, 2H, CH_2_N), 2.79 (s, 3H, ArCH_3_), 2.30 (s, 3H, COCH_3_), 1.53–1.69 (m, 4H, 2 × NCH_2_CH_2_), 1.27 (m, 2H, NHCH_2_CH_2_CH_2_); MS (ESI) *m*/*z* = 488 [M+H]^+^; Anal. Calcd. for C_28_H_31_N_4_O_4_: C, 68.97; H, 6.41; N, 11.49; Found: C, 68.82; H, 6.53; N, 11.63.

### 1-(4-Methoxyphenyl)-*N*-(5-(2-methylbenzamido)pentyl)-9H-pyrido[3,4-b]indole-3-carboxamide (7i)

Refer to the synthesis of **7a**, the title product was obtained from **5i** and acetylsalicylic acid to afford a pale yellow solid 4.2 g, yield: 73%. Analytical data for **7i**: IR (KBr, cm^−1^): 3400, 2961, 1677, 1574, 1484, 1428, 1321, 1264, 1020. Analytical data for **7i**: ^1^H NMR (CDCl_3_, 300 MHz): *δ* 9.76 (s, 1H, NH), 8.71–8.75 (m, 2H, Ar-H, CONH), 8.15 (d, 1H, *J* = 7.5 Hz, Ar-H), 8.06 (m, 1H, Ar-H), 7.85 (d, 2H, *J* = 7.5 Hz, Ar-H), 7.56–7.63 (m, 3H, Ar-H), 7.41–7.47 (m, 3H, Ar-H), 7.05 (d, 2H, *J* = 7.5 Hz, Ar-H), 3.85 (s, 3H, OCH_3_), 3.59 (m, 2H, NCH_2_). 3.44 (m, 2H, NCH_2_), 2.31 (s, 3H, CH_3_CO), 1.70–1.85 (m, 4H, 2 ×NCH_2_CH_2_), 1.28 (m, 2H, NCH_2_CH_2_CH_2_). MS (ESI) *m*/*z* = 580 [M+H]^+^; Anal. Calcd. for C_34_H_35_N_4_O_5_: C, 70.45; H, 6.09; N, 9.67; Found: C, 70.32; H, 5.92; N, 9.52.

### *N*-(6-(2-Methylbenzamido)hexyl)-9H-pyrido[3,4-b]indole-3-carboxamide (7j)

Refer to the synthesis of **7a**, the title product was obtained from **5j** and acetylsalicylic acid to afford a pale yellow solid 3.8 g, yield: 77%. Analytical data for **7j**: IR (KBr, cm^−1^): 3466, 2960, 1650, 1574, 1483, 1436, 1300, 1267, 1028. Analytical data for **7j**: ^1^H NMR (CDCl_3_, 300 MHz): *δ* 11.08 (s, 1H, NH), 8.89 (s, 1H, Ar-H), 8.70 (s, 1H, Ar-H), 8.38 (m, 1H, CONH), 8.12–8.17 (m, 2H, CONH, Ar-H), 8.03 (m, 1H, Ar-H), 7.77 (m, 1H, Ar-H), 7.53–7.60 (m, 2H, Ar-H), 7.36–7.47 (m, 3H, Ar-H), 3.55 (m, 2H, NCH_2_), 3.41 (m, 2H, CH_2_N), 2.28 (m, 3H, CH_3_CO), 1.53–1.68 (m, 2H, NCH_2_CH_2_), 1.23–1.40 (m, 4H, 2 × NHCH_2_CH_2_CH_2_); MS (ESI) *m*/*z* = 488 [M+H]^+^; Anal. Calcd. for C_28_H_31_N_4_O_4_: C, 68.97; H, 6.41; N, 11.49; Found: C, 68.82; H, 6.53; N, 11.36.

### 1-Methyl-*N*-(6-(2-methylbenzamido)hexyl)-9H-pyrido[3,4-b]indole-3-carboxamide (7k)

Refer to the synthesis of **7a**, the title product was obtained from **5k** (3.2 g, 10 mmol) and acetylsalicylic acid (1.8 g, 10 mmol) to afford a pale yellow solid 3.6 g, yield: 73%. Analytical data for **7k**: IR (KBr, cm^−1^): 3451, 2925, 1669, 1576, 1475, 1427, 1316, 1265, 1028. ^1^H NMR (CDCl_3_, 300 MHz): *δ* 8.78 (s, 1H, Ar-H), 8.67 (s, 1H, CONH), 8.10 (m, 1H, Ar-H), 7.90 (m, 1H, Ar-H), 7.53–7.66 (m, 3H, Ar-H), 7.29–7.35 (m, 2H, Ar-H), 7.06 (m, 1H, Ar-H), 3.53–3.59 (m, 2H, NCH_2_), 3.36–3.40 (m, 2H, CH_2_N), 2.81 (s, 3H, ArCH_3_), 2.30 (s, 3H, COCH_3_), 1.59–1.70 (m, 4H, 2 × NCH_2_CH_2_), 1.25–1.46 (m, 4H, 2 × NHCH_2_CH_2_CH_2_); MS (ESI) *m*/*z* = 502 [M+H]^+^; Anal. Calcd. for C_29_H_33_N_4_O_4_: C, 69.44; H, 6.63; N, 11.17; Found: C, 69.26; H, 6.75; N, 11.32.

### 1-(4-Methoxyphenyl)-*N*-(6-(2-methylbenzamido)hexyl)-9H-pyrido[3,4-b]indole-3-carboxamide (7l)

Refer to the synthesis of **7a**, the title product was obtained from **5l** and acetylsalicylic acid to afford a pale yellow solid 4.2 g, yield: 70%. Analytical data for **7l**: IR (KBr, cm^−1^): 3451, 2965, 1651, 1514, 1463, 1403, 1289, 1229, 1021. ^1^H NMR (CDCl_3_, 300 MHz): *δ* 8.67–8.73 (m, 2H, CONH, Ar-H), 8.12 (m, 1H, Ar-H), 8.06 (m, 1H, Ar-H), 7.90 (d, 2H, *J* = 9.0 Hz, Ar-H), 7.61–7.73 (m, 3H, Ar-H), 7.43–7.56 (m, 2H, Ar-H), 7.29 (m, 1H, Ar-H), 7.07 (d, 2H, *J* = 9.0 Hz, Ar-H), 3.89 (s, 3H, OCH_3_), 3.60 (m, 2H, NCH_2_), 3.42 (m, 2H, CH_2_N), 2.30 (s, 3H, COCH_3_), 1.59–1.70 (m, 4H, 2 × NCH_2_CH_2_), 1.23–1.45 (m, 4H, 2 × NCH_2_CH_2_CH_2_); MS (ESI) *m*/*z* = 594 [M+H]^+^; Anal. Calcd. for C_35_H_37_N_4_O_5_: C, 70.81; H, 6.28; N, 9.44; Found: C, 70.69; H, 6.49; N, 9.33.

### *N*-(3-(2-Hydroxybenzamido)propyl)-9H-pyrido[3,4-b]indole-3-carboxamide (8a)

To a solution of **7a** (2.2 g, 5.0 mmol) in 20 mL methanol was added NaOH (0.4 g, 10.0 mmol) in 5 mL water and the mixture was stirred at 60 °C for 2 h, then added 10 mL water, neutralized with 2 M HCl and extracted with ethyl acetate (3 × 50 mL). The organic layer was washed with brine, dried over Na_2_SO_4_, and concentrated in vacuo. The residue was purified by silica gel column chromatography (petroleum ether: ethyl acetate = 1:1 to 1:3, v/v) to to give a pale yellow solid 1.7 g, yield: 90%. Purity: 97.2% (by HPLC). Analytical data for **8a**: ^1^H NMR (CDCl_3_, 300 MHz): *δ* 11.39 (s, 1H, NH), 8.78 (s, 1H, Ar-H), 8.61 (s, 1H, Ar-H), 8.49 (s, 1H, CONH), 8.10 (m, 2H, Ar-H), 7.90 (m, 1H, Ar-H), 7.44–7.62 (m, 3H, Ar-H), 7.30 (s, 1H, Ar-H), 7.05 (m, 1H, Ar-H), 3.53–3.72 (m, 4H, 2 × NCH_2_), 1.90–1.95 (m, 2H, NCH_2_CH_2_); MS (ESI) *m*/*z* = 389 [M+H]^+^; HRMS (ESI): *m*/*z* calcd for C_22_H_21_N_4_O_3_: 389.1614; found: 389.1630 [M+H]^+^.

### *N*-(3-(2-Hydroxybenzamido)propyl)−1-methyl-9H-pyrido[3,4-b]indole-3-carboxamide (8b)

Refer to the synthesis of **8a**, the title product was obtained from **7b** to afford a pale yellow solid 1.8 g, yield: 88%. Purity: 98.2% (by HPLC). Analytical data for **8b**: ^1^H NMR (CDCl_3_, 300 MHz): *δ* 8.87 (s, 1H, Ar-H), 8.73 (s, 1H, Ar-H), 8.48 (s, 1H, NH), 8.14 (d, 1H, *J* = 6.0 Hz, Ar-H), 8.06 (m, 1H, Ar-H), 7.56 (m, 2H, Ar-H), 7.34 (m, 2H, Ar-H), 7.05 (m, 1H, Ar-H), 3.71 (m, 2H, CH_2_N), 3.56 (m, 2H, NCH_2_), 2.83 (s, 3H, ArCH_3_), 1.91 (m, 2H, NCH_2_CH_2_). MS (ESI) *m*/*z* = 403 [M+H]^+^; HRMS (ESI): *m*/*z* calcd for C_23_H_23_N_4_O_3_: 403.1770; found: 403.1787 [M+H]^+^.

### *N*-(3-(2-Hydroxybenzamido)propyl)-1-(4-methoxyphenyl)-9H-pyrido[3,4-b]indole-3-carboxamide (8c)

Refer to the synthesis of **8a**, the title product was obtained from **7c** to afford a pale yellow solid 2.2 g, yield: 88%. Purity: 98.1% (by HPLC). Analytical data for **8c**: ^1^H NMR (CDCl_3_, 300 MHz): *δ* 12.45 (s, 1H, NH), 8.77 (s, 1H, Ar-H), 8.68 (s, 1H, NH), 8.60 (s, 1H, CONH), 8.22–8.30 (m, 3H, Ar-H), 8.11 (m, 1H, Ar-H), 7.90 (m, 1H, Ar-H), 7.48–7.56 (m, 3H, Ar-H), 7.33–7.39 (m, 3H, Ar-H), 7.13 (m, 1H, Ar-H), 6.99 (m, 1H, Ar-H), 3.84 (s, 3H, OCH_3_), 3.63 (m, 2H, CH_2_N), 3.51 (m, 2H, NCH_2_), 2.01 (m, 2H, NCH_2_CH_2_). MS (ESI) *m*/*z* = 495 [M+H]^+^; HRMS (ESI): *m*/*z* calcd for C_29_H_27_N_4_O_4_: 495.2032; found: 495.2046 [M+H]^+^.

### *N*-(4-(2-Hydroxybenzamido)butyl)-9H-pyrido[3,4-b]indole-3-carboxamide (8d)

Refer to the synthesis of **8a**, the title product was obtained from **7d** (2 g, 10 mmol) to afford a pale yellow solid 2.0 g, yield: 88%. Purity: 97.3% (by HPLC). Analytical data for **8d**: ^1^H NMR (CDCl_3_, 500 MHz): *δ* 11.57 (s, 1H, NH), 9.00 (s, 1H, CONH), 8.88 (s, 1H, Ar-H), 8.77 (s, 1H, Ar-H), 8.30 (m, 1H, Ar-H), 7.18 (d, 1H, *J* = 5.0 Hz, Ar-H), 7.54–7.61 (m, 3H, Ar-H), 7.33 (m, 1H, Ar-H), 7.17 (s, 1H, Ar-H), 7.03 (m, 1H, Ar-H), 3.57–3.61 (m, 4H, 2 × NCH_2_), 1.45–1.68 (m, 4H, 2 × NCH_2_CH_2_); MS (ESI) *m*/*z* = 403 [M+H]^+^; HRMS (ESI): *m*/*z* calcd for C_23_H_23_N_4_O_3_: 403.1770; found: 403.1791 [M+H]^+^.

### *N*-(4-(2-Hydroxybenzamido)butyl)−1-methyl-9H-pyrido[3,4-b]indole-3-carboxamide (8e)

Refer to the synthesis of **8a**, the title product was obtained from **7e** (2.4 g, 10 mmol) to afford a pale yellow solid 1.8 g, yield: 87%. Purity: 96.5% (by HPLC). Analytical data for **8e**: ^1^H NMR (CDCl_3_, 300 MHz): *δ* 11.92 (s, 1H, NH), 8.66 (s, 1H, CONH), 8.58 (m, 1H, Ar-H), 8.33 (m, 1H, Ar-H), 7.58–7.65 (m, 4H, Ar-H), 7.28 (m, 1H, Ar-H), 6.83 (m, 1H, Ar-H), 3.06–3.18 (m, 4H, 2 × NCH_2_), 2.85 (s, 3H, ArCH_3_), 1.43–1.59 (m, 4H, 2 × NCH_2_CH_2_); MS (ESI) *m*/*z* = 417 [M+H]^+^; HRMS (ESI): *m*/*z* calcd for C_24_H_25_N_4_O_3_: 417.1927; found: 417.1918 [M+H]^+^.

### *N*-(4-(2-Hydroxybenzamido)butyl)-1-(4-methoxyphenyl)-9H-pyrido[3,4-b]indole-3-carboxamide (8f)

Refer to the synthesis of **8a**, the title product was obtained from **7f** (2.8 g, 10 mmol) to afford a pale yellow solid 2.3 g, yield: 90%. Purity: 95.4% (by HPLC). Analytical data for **8f**: ^1^H NMR (CDCl_3_, 300 MHz): *δ* 12.22 (s, 1H, NH), 8.67 (s, 1H, Ar-H), 8.51 (s, 1H, CONH), 8.17 (m, 2H, Ar-H), 8.02 (s, 1H, Ar-H), 7.59 (m, 2H, Ar-H), 7.33–7.46 (m, 4H, Ar-H), 7.13 (m, 2H, Ar-H), 6.91 (m, 1H, Ar-H), 3.86 (s, 3H, OCH_3_), 3.24–3.39 (m, 4H, 2 × NCH_2_), 1.61 (m, 4H, 2 × NCH_2_CH_2_). MS (ESI) *m*/*z* = 509 [M+H]^+^; HRMS (ESI): *m*/*z* calcd for C_30_H_29_N_4_O_4_: 509.2189; found: 509.2208 [M+H]^+^.

### *N*-(5-(2-Hydroxybenzamido)pentyl)-9H-pyrido[3,4-b]indole-3-carboxamide (8g)

Refer to the synthesis of **8a**, the title product was obtained from **7g** (2.4 g, 10 mmol) to afford a pale yellow solid 1.9 g, yield: 91%. Purity: 95.4% (by HPLC). Analytical data for **8g**: ^1^H NMR (CDCl_3_, 500 MHz): *δ* 12.01 (s, 1H, NH), 8.86 (s, 1H, Ar-H), 8.66–8.72 (m, 2H, NH, Ar-H), 8.37 (s, 1H, Ar-H), 7.18 (d, 1H, *J* = 6.0 Hz, Ar-H), 7.55–7.68 (m, 4H, Ar-H), 7.16–7.22 (m, 3H, NH, Ar-H), 3.40–3.72 (m, 4H, 2 × NCH_2_), 1.45–1.65 (m, 4H, 2 × NCH_2_CH_2_), 1.21–1.27 (m, 2H, NCH_2_CH_2_CH_2_); MS (ESI) *m*/*z* = 417 [M+H]^+^ ; HRMS (ESI): *m*/*z* calcd for C_24_H_25_N_4_O_3_: 417.1927; found: 417.1944 [M+H]^+^.

### *N*-(5-(2-Hydroxybenzamido)pentyl)-1-methyl-9H-pyrido[3,4-b]indole-3-carboxamide (8h)

Refer to the synthesis of **8a**, the title product was obtained from **7h** (2.4 g, 10 mmol) to afford a pale yellow solid 2.0 g, yield: 92%. Purity: 96.0% (by HPLC). Analytical data for **8h**: ^1^H NMR (CDCl_3_, 300 MHz): *δ* 8.78 (s, 1H, NH), 8.39 (s, 1H, NH), 8.17 (m, 1H, Ar-H), 8.02 (m, 1H, Ar-H), 7.58–7.60 (m, 2H, Ar-H), 7.36 (m, 1H, Ar-H), 7.19 (m, 2H, Ar-H), 7.05 (m, 1H, Ar-H), 3.58–3.72 (m, 4H, 2 × NCH_2_), 2.87 (s, 3H, ArCH_3_), 1.53 (m, 4H, 2 × NCH_2_CH_2_), 1.18 (m, 2H, NHCH_2_CH_2_CH_2_); MS (ESI) *m*/*z* = 431 [M+H]^+^; HRMS (ESI): *m*/*z* calcd for C_25_H_27_N_4_O_3_: 431.2083; found: 431.2102 [M+H]^+^.

### *N*-(5-(2-Hydroxybenzamido)pentyl)-1-(4-methoxyphenyl)-9H-pyrido[3,4-b]indole-3-carboxamide (8i)

Refer to the synthesis of **8a**, the title product was obtained from **7i** (2.9 g, 10 mmol) to afford a pale yellow solid 2.3 g, yield: 89%. Purity: 98.4% (by HPLC). Analytical data for **8i**: ^1^H NMR (CDCl_3_, 300 MHz): *δ* 12.60 (s, 1H, NH), 8.76 (m, 1H, Ar-H), 8.62 (m, 1H, Ar-H), 8.13–8.24 (m, 2H, CONH, Ar-H), 8.02 (s, 1H, Ar-H), 7.79 (m, 1H, Ar-H), 7.43–7.58 (m, 5H, Ar-H), 7.09 (m, 2H, Ar-H), 6.93 (m, 1H, Ar-H), 3.91 (s, 3H, OCH_3_), 3.55–3.65 (m, 4H, 2 × NCH_2_), 1.78–1.84 (m, 4H, 2 × NCH_2_CH_2_), 1.24 (m, 2H, NHCH_2_CH_2_CH_2_); MS (ESI) *m*/*z* = 523 [M+H]^+^; HRMS (ESI): *m*/*z* calcd for C_31_H_31_N_4_O_4_: 523.2345; found: 523.2328 [M+H]^+^.

### *N*-(6-(2-Hydroxybenzamido)hexyl)-9H-pyrido[3,4-b]indole-3-carboxamide (8j)

Refer to the synthesis of **8a**, the title product was obtained from **7j** (2.4 g, 10 mmol) to afford a pale yellow solid 1.8 g, yield: 87%. Purity: 95.4% (by HPLC). Analytical data for **8j**: ^1^H NMR (CDCl_3_, 300 MHz): *δ* 11.35 (s, 1H, NH), 8.86 (s, 1H, Ar-H), 8.77 (s, 1H, Ar-H), 8.57 (m, 2H, CONH, Ar-H), 8.15 (m, 1H, Ar-H), 7.78 (m, 1H, Ar-H), 7.45–7.67 (m, 3H, Ar-H), 7.13 (m, 1H, Ar-H), 6.91 (m, 1H, Ar-H), 3.52–3.71 (m, 4H, 2 × NCH_2_), 1.60–1.77 (m, 4H, 2 × NCH_2_CH_2_), 1.19–1.32 (m, 4H, 2 × NHCH_2_CH_2_CH_2_); MS (ESI) *m*/*z* = 431 [M+H]^+^; HRMS (ESI): *m*/*z* calcd for C_25_H_27_N_4_O_3_: 431.2083; found: 431.2098 [M+H]^+^.

### *N*-(6-(2-Hydroxybenzamido)hexyl)-1-methyl-9H-pyrido[3,4-b]indole-3-carboxamide (8k)

Refer to the synthesis of **8a**, the title product was obtained from **7k** (2.5 g, 10 mmol) to afford a pale yellow solid 1.9 g, yield: 87%. Purity: 98.5% (by HPLC). Analytical data for **8k**: ^1^H NMR (CDCl_3_, 500 MHz): *δ* 11.87 (s, 1H, NH), 8.66 (m, 1H, Ar-H), 8.56 (m, 1H, Ar-H), 8.35 (d, 1H, *J* = 10.0 Hz, Ar-H), 7.62–7.76 (m, 4H, Ar-H), 7.31 (m, 1H, Ar-H), 6.98 (m, 2H, CONH, Ar-H), 3.36 (m, 2H, NCH_2_), 2.85 (s, 3H, ArCH_3_), 3.02 (m, 2H, NCH_2_), 1.53–1.66 (m, 4H, 2 × NCH_2_CH_2_), 1.23–1.41 (m, 4H, 2 × NHCH_2_CH_2_CH_2_); MS (ESI) *m*/*z* = 445 [M+H]^+^; HRMS (ESI): *m*/*z* calcd for C_26_H_29_N_4_O_3_: 445.2240; found: 445.2223 [M+H]^+^.

### *N*-(6-(2-Hydroxybenzamido)hexyl)-1-(4-methoxyphenyl)-9H-pyrido[3,4-b]indole-3-carboxamide (8l)

Refer to the synthesis of **8a**, the title product was obtained from **7l** (3.0 g, 10 mmol) to afford a pale yellow solid 2.4 g, yield: 88%. Purity: 96.2% (by HPLC). Analytical data for **8l**: ^1^H NMR (CDCl_3_, 300 MHz): *δ* 11.80 (s, 1H, NH), 10.43 (s, 1H, NH), 8.77 (m, 3H, Ar-H, NH), 8.40 (d, 1H, *J* = 6.0 Hz, Ar-H), 8.17 (d, 2H, *J* = 6.3 Hz, Ar-H), 7.57–7.70 (m, 3H, Ar-H), 7.19–7.32 (m, 4H, Ar-H), 6.85 (m, 1H, Ar-H), 3.89 (s, 3H, OCH_3_), 3.35–3.63 (m, 4H, 2 × NCH_2_), 1.61–1.83 (m, 4H, 2 × NCH_2_CH_2_), 1.22–1.38 (m, 4H, 2 × NHCH_2_CH_2_CH_2_); MS (ESI) *m*/*z* = 537 [M+H]^+^; HRMS (ESI): *m*/*z* calcd for C_32_H_33_N_4_O_4_: 537.2502; found: 537.2488 [M+H]^+^.

### *N*-(5-acetamidopentyl)-1-methyl-9H-pyrido[3,4-b]indole-3-carboxamide (10a)

To a solution of **5h** (0.31 g, 1.0 mmol) and triethylamine (0.15 g, 1.5 mmol) in 3 mL anhydrous DMF was slowly added acetyl chloride (86 mg, 1.1 mmol) in 1 mL anhydrous CH_2_Cl_2_ under ice bath. After addition, the mixture was stirred at room temperature for 2 h, and then poured into 30 mL water, then extracted with ethyl acetate (3 × 25 mL). The combined organic layer was washed with brine, dried with Na_2_SO_4_, and concentrated in vacuo. The residue was purified by silica gel column chromatography (petroleum ether: ethyl acetate = 1:1 to 1:3, v/v) to afford a pale yellow solid, yield :79%. Purity: 95.6% (by HPLC). Analytical data for **10a**: IR (KBr, cm^−1^): 3432, 2941, 1628, 1476, 1420, 1250, 1018. ^1^H NMR (CDCl_3_, 300 MHz): *δ* 11.91 (s, 1H, NH), 8.63–8.66 (m, 2H, Ar-H, NH), 8.35 (s, 1H, Ar-H), 7.63 (d, 1H, *J* = 6.0 Hz, Ar-H), 7.59 (m, 1H, Ar-H), 7.30 (m, 1H, Ar-H), 5.76 (s, 1H, NH), 3.66 (t, 2H, *J* = 6.0 Hz, CONHCH_2_), 3.40 (m, 2H, CH_2_N), 2.84 (s, 3H, Ar-CH_3_), 2.33 (s, 3H, COCH_3_), 1.69–1.82 (m, 4H, 2 × NCH_2_CH_2_), 1.23–1.30 (m, 2H, NCH_2_CH_2_CH_2_); MS (ESI) *m*/*z* = 353 [M+H]^+^.

### *N*-(5-benzamidopentyl)-1-methyl-9H-pyrido[3,4-b]indole-3-carboxamide (10b)

Refer to the synthesis of **10a**, the title product was obtained from **5h** (0.31 g, 1.0 mmol) and benzoyl chloride (0.15 g, 1.1 mmol) to afford a pale yellow solid, yield: 75%. Purity: 98.0% (by HPLC). Analytical data for **10b**: IR (KBr, cm^−1^): 3466, 2958, 1620, 1468, 1418, 1244, 1011. ^1^H NMR (CDCl_3_, 300 MHz): *δ* 11.91 (s, 1H, NH), 8.67 (s, 1H, Ar-H), 8.61 (s, 1H, Ar-H), 8.49 (m, 1H, NHCO), 8.34 (m, 1H, Ar-H), 7.84 (d, 1H, *J* = 6.0 Hz, Ar-H), 7.64 (d, 1H, *J* = 6.0 Hz, Ar-H), 7.51–7.58 (m, 2H, Ar-H), 7.44 (m, 2H, Ar-H), 7.28 (m, 1H, Ar-H), 3.40 (m, 2H, NCH_2_), 3.31 (m, 2H, NCH_2_), 2.84 (s, 3H, CH_3_), 1.65–1.78 (m, 4H, 2 × NCH_2_CH_2_), 1.22–1.28 (m, 2H, NCH_2_CH_2_CH_2_); MS (ESI) *m*/*z* = 415 [M+H]^+^.

### Biological evaluation

#### MTT assay

Hepatocellular carcinoma cells (SMMC-7721 and Hep G2), Human colon cancer cell lines (HCT116), human bladder carcinoma cells (EJ) or human lung cancer cells (H460) at 10^4^ cells per well were cultured in 10% FBS DMEM in 96-well flat-bottom microplates overnight. The cells were incubated in triplicate with, or without, different concentrations of each test compound for 48 h. During the last 4 h incubation, 30 μL of tetrazolium dye (MTT) solution (5 mg mL^−1^) was added to each well. The resulting MTT-formazan crystals were dissolved in 150 μL DMSO, and absorbance was measured spectrophotometrically at 570 nm using an ELISA plate reader. The inhibition induced by each test compound at the indicated concentrations was expressed as a percentage. The concentration required for 50% inhibition (IC_50_) was calculated using the software (GraphPadPrism Version 4.03).

#### Flow cytometry assay of cell apoptosis

SMMC-7721 cells were cultured overnight and incubated in triplicate with compound **8h** (7.0 and 14 μM), 5-FU (14 μM), or vehicle for 48 h. The cells were harvested, and stained with FITC-Annexin V and PI (BioVision) at room temperature for 15 min. The percentage of apoptotic cells was determined by flow cytometry (Beckman Coulter) analysis.

#### Mitochondrial membrane potential assay

Mitochondrial membrane potential was assessed using a Beyotime (China) kit with JC-1. Briefly, the SMMC-7721 cells in the logarithmic growth phase were treated with **8h** (7.0 or 14 μM), 5-FU (14 μM), or vehicle control for 24 h. The cells were harvested, and then labeled with JC-1 in accordance with the manufacturer’s instructions. The samples labeled with JC-1 were analyzed via flow cytometry. For the detection of JC-1, excitation was set at 530 nm, and emissions collected at 585 nm.

#### Western blot assay

The mechanisms of the cell apoptosis were determined by western blot assay. SMMC-7721 cells at 1.5 × 10^5^/mL were treated with 3.5, 7.0, or 14 μM **8h** or vehicle control for 8 h. After harvested and lyzed, the cell lysates (50 μg/lane) were separated by SDS-PAGE (12% gel) and transferred onto nitrocellulose membranes. After blocked with 5% fat-free milk, the target proteins were probed with anti-Bcl-2, anti-Bax, anti-caspase-3, anti-PARP, and anti-β-actin antibodies (Cell Signaling, Boston), respectively. The bound antibodies were detected by HRP-conjugated second antibodies and visualized using the enhanced chemiluminescent reagent.

## Additional Information

**How to cite this article**: Xu, Q.-B. *et al*. Design, synthesis and biological evaluation of hybrids of β-carboline and salicylic acid as potential anticancer and apoptosis inducing agents. *Sci. Rep.* **6**, 36238; doi: 10.1038/srep36238 (2016).

**Publisher’s note:** Springer Nature remains neutral with regard to jurisdictional claims in published maps and institutional affiliations.

## Figures and Tables

**Figure 1:**
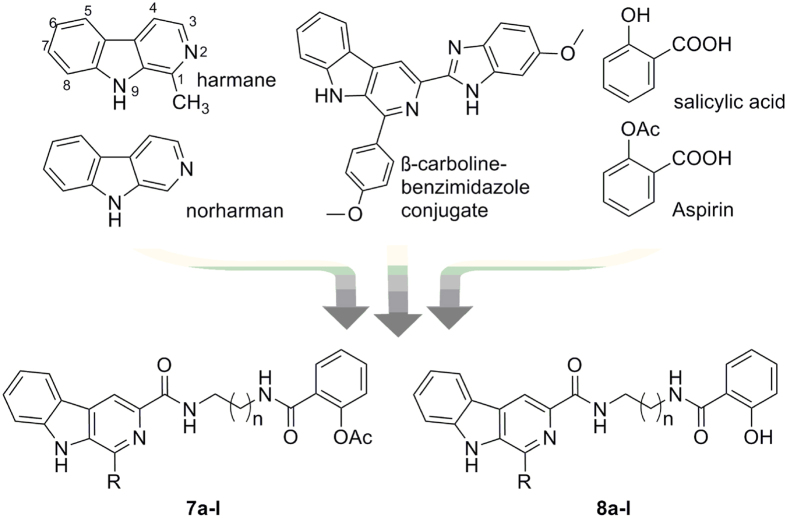
The design of novel β-carbolines/salicylic acid hybrids.

**Table 1:** The IC_50_ values of synthetic compounds 7a-l, 8a-l, and 10a-b against five human cancer cell lines.

Compd.	R	n	*In vitro* cytotoxicity (IC_50_, μM)^a^				
			SMMC-7721	Hep G2	HCT116	EJ	H460
5-FU	/	/	28.7	35.2	19.6	ND^b^	ND
harmine	/	/	49.1	55.3	46.7	ND	ND
**7a**	H	2	34.7	32.0	36.0	25.3	31.1
**7b**	Me	2	27.3	23.7	28.9	23.2	30.5
**7c**	Ph-4-OMe	2	>50	>50	>50	>50	>50
**7d**	H	3	24.3	19.1	21.4	18.4	23.2
**7e**	Me	3	17.8	15.2	14.8	13.3	20.1
**7f**	Ph-4-OMe	3	25.6	31.2	32.6	29.4	38.4
**7g**	H	4	15.9	14.3	17.1	15.3	19.4
**7h**	Me	4	12.8	11.3	15.8	12.1	13.2
**7i**	Ph-4-OMe	4	38.2	>50	45.2	>50	27.5
**7j**	H	5	23.3	28.0	26.6	18.2	15.3
**7k**	Me	5	11.3	16.9	15.3	13.7	14.3
**7l**	Ph-4-OMe	5	>50	ND	>50	ND	>50
**8a**	H	2	22.6	18.9	20.1	15.9	25.5
**8b**	Me	2	19.1	ND	13.6	15.3	22.2
**8c**	Ph-4-OMe	2	36.0	45.8	33.2	38.6	>50
**8d**	H	3	13.0	11.7	15.9	12.1	15.2
**8e**	Me	3	7.93	9.26	9.36	9.01	11.9
**8f**	Ph-4-OMe	3	20.1	17.7	18.2	15.0	22.8
**8g**	H	4	10.7	9.12	9.18	8.71	12.5
**8h**	Me	4	6.97	7.12	8.25	7.89	13.1
**8i**	Ph-4-OMe	4	46.8	30.8	>50	>50	38.5
**8j**	H	5	13.4	17.2	18.6	16.5	21.5
**8k**	Me	5	10.1	13.6	10.7	13.2	11.4
**8l**	Ph-4-OMe	5	>50	42.0	>50	ND	>50
**10a**	/	/	>50	>50	>50	ND	ND
**10b**	/	/	>50	>50	>50	ND	ND

^a^The inhibitory effects of individual compounds on the proliferation of cancer cell lines were determined by the MTT assay. The data are the mean values of IC_50_ from at least three independent experiments.

^b^Not detected.
